# Measuring outcomes after major abdominal surgery during hospitalization: reliability and validity of the Postoperative Morbidity Survey

**DOI:** 10.1186/2047-0525-2-1

**Published:** 2013-02-04

**Authors:** Simon J Davies, James Francis, Jonathan Dilley, R Jonathan T Wilson, Simon J Howell, Victoria Allgar

**Affiliations:** 1 Department of Anaesthesia, York Hospitals NHS Foundation Trust, Wigginton Road, York YO31 8HE, UK; 2 Academic Unit of Anaesthesia, Leeds General Infirmary, Great George Street, Leeds, LS1 3EX, UK; 3 Hull York Medical School, The University of York, Heslington, York, YO10 5DD, UK

**Keywords:** Major surgery, Morbidity, Postoperative morbidity survey, Postoperative outcome

## Abstract

**Background:**

Measurement of outcomes after major abdominal surgery has traditionally focused on mortality, however the low incidence in elective surgery makes this measure a poor comparator. The Postoperative Morbidity Survey (POMS) prospectively assesses short-term morbidity, and may have clinical utility both as a core outcome measure in clinical trials and quality of care. The POMS has been shown to be a valid outcome measure in a mixed surgical population, however it has not been studied in patients undergoing major abdominal surgery. This study assessed the inter-rater reliability and validity of the POMS in patients undergoing major abdominal surgery.

**Methods:**

Patients undergoing elective major abdominal surgery were visited on postoperative day 1 until discharge by two novice observers who administered the POMS in order to assess inter-rater reliability. Subjects who had previously had the POMS performed prospectively on postoperative days 3 and 5 were identified from a database. The pattern and prevalence of morbidity was analyzed against hospital length of stay (LOS) in order to validate the POMS in this patient group.

**Results:**

Fifty one patients were recruited to the inter-rater reliability study giving a total of 263 POMS assessments. Inter-rater reliability showed a 97.7% agreement with a κ coefficient of 0.912 (95% CI: 0.842 to 0.982). On domain analysis percentage agreement was lowest in the gastrointestinal domain (87.5%), whilst correlation was lowest in the wound (κ: 0.04; 95% CI: −1.0 to 1.0) and hematological domains (κ: 0.378; 95% CI: 0.035 to 0.722). All other domains showed at least substantial agreement. POMS assessments were analyzed for postoperative days 3 (n = 258) and 5 (n = 362). The absence or presence of morbidity as measured by the POMS was associated with a hospital LOS of 6 (IQR: 4 to 7) *vs.* 11 (IQR: 8 to 15) days on postoperative day 3 (*P* <0.0001), and 7 (IQR: 6 to 10) *vs.* 13 (IQR: 9 to 19) days on postoperative day 5 (*P* <0.0001). The presence of any morbidity on postoperative day 5 conferred an odds ratio for a prolonged hospital LOS of 11.9 (95% CI: 5.02 to 11.92).

**Conclusions:**

This study shows that the POMS is both a reliable and valid measure of short-term postoperative morbidity in patients undergoing major abdominal surgery.

## Background

Measurement of patient outcomes after major abdominal surgery has traditionally focused on mortality; however, the relatively low event rate for the majority of elective surgical procedures makes this a poor discriminator of both the quality of care between institutions and the effectiveness of interventions. Although absolute in terms of an outcome measure, the use of mortality as a clinical end-point is not without its discrepancies. The temporal variation in reporting, ranging from in hospital mortality to up to 1 year mortality, means that it is a difficult and poor comparator, and the loss of patients to long-term follow-up is an additional confounder. It has been suggested that 30-day mortality may underestimate short-term patient outcomes following major abdominal surgery, with a significant increase in mortality seen at 90 days [1].

Morbidity is a more frequent occurrence after major surgery, particularly in the higher risk patient. The importance of complications in the immediate postoperative period is clear, however perioperative complications may have long-term implications for both the quality and quantity of an individual’s life with an associated reduction in median survival [1,2]. The measurement and reporting of outcomes following major surgery is a fundamental process to improving the quality and safety of care that a health system provides, both in terms of the health framework and delivery of care by institutions and the individual clinicians themselves [3,4]. It is theoretically desirable to have a core set of outcomes measured and reported as a minimum in clinical research, allowing differing interventions to be compared and contrasted with greater ease and reducing the risk of outcome reporting bias.

The postoperative morbidity survey (POMS) is a nine-domain system that prospectively identifies short-term mortality after surgery [5]. For each of the nine domains morbidity is recorded on the presence or absence of preset criteria and it appears to accurately describe the pattern and prevalence of morbidity in the postoperative setting.

The purpose of this study was firstly to ascertain whether it is feasible to use the POMS as a core outcome measure for reporting in clinical trials by assessing the inter-rater reliability of two novice observers, and secondly to evaluate its validity as an outcome measure in terms of its prediction of hospital length of stay (LOS).

## Methods

The protocol was approved by the Local Research Ethics Committee of York Hospital NHS Trust. A member of the research team screened patients for eligibility, and written informed consent was obtained from patients prior to surgery. The study was performed in 2 parts, a prospective analysis of the inter-rater agreement of the POMS, and a retrospective analysis of data to assess its validity.

### Inter-rater agreement of the POMS

#### Inclusion criteria

Patients undergoing elective major abdominal surgery defined as procedures expected to last more than 2 hours, or with an anticipated blood loss greater than 500 mL, were recruited. This included general surgery (colorectal, pancreatic, gastric surgery), vascular surgery (abdominal aortic aneurysm repair) and urological surgery (cystectomy, prostatectomy and nephrectomy).

#### Protocol

Two independent and novice researchers, neither of whom had used the POMS previously, visited patients independently on postoperative day 1 and then daily until hospital discharge. The researchers observed and questioned the patient regarding the items defined in the POMS (Table 1), and also reviewed patient notes, charts and pathology results in order to complete the POMS accurately.

**Table 1:** The Postoperative Morbidity Survey (POMS) showing morbidity types, definitions and source data

**Morbidity type**	**Criteria**	**Source data**
Pulmonary	*De novo* requirement for supplemental oxygen or other respiratory support (e.g., CPAP or IPPV)	Patient observation
		Treatment chart
		Observation chart
Infectious	Currently on antibiotics or temperature >38°C in the last 24 hrs	Treatment chart
		Observation chart
Renal	Presence of oliguria (<500 mL/day), increased serum creatinine (>30% from baseline value), or urinary catheter in place for a non-surgical reasons	Patient observation
		Fluid balance chart
		Biochemistry results
Gastrointestinal	Unable to tolerate an enteral diet (either by mouth or feeding tube) for any reason, including nausea, vomiting and abdominal distension	Patient questioning
		Fluid balance chart
Cardiovascular	Diagnostic test or therapy in last 24 hrs for any of the following reasons: *de novo* myocardial infarction or ischemia, hypotension (requiring drug therapy or fluid >200 mL/hr), atrial or ventricular arrhythmia or pulmonary edema	Treatment chart
		Fluid chart
		Observation chart
		Note review
Neurological	Presence of a *de novo* focal deficit, coma or confusion/delirium	Patient questioning
		Note review
Wound complications	Wound dehiscence requiring surgical exploration or drainage or pus from the wound	Note review
		Pathology results
Hematological	Requirement for any of the following within last 24 hrs: blood, platelets, fresh frozen plasma or cryoprecipitate	Fluid balance chart
Pain	Surgical wound pain significant enough to require parenteral opiates or regional anesthesia	Treatment chart
		Patient questioning

#### Statistical analysis

Inter-rater reliability of the individual components of the POMS (absence or presence of individual domain morbidities) and the occurrence of any morbidity in any domain was assessed using the κ coefficient, which assesses agreement beyond that expected by chance alone. Non-parametric data was analyzed using the Mann–Whitney U-test. Incidences were calculated using the χ^2^ test. Relationships between two categorical variables were analyzed by Fisher’s exact test, and strength of association calculated. A *P* value less than 0.05 was considered significant. Where appropriate 95% confidence intervals (CI) were calculated. POMS domains that were significant on univariate analysis were entered into a multivariate model for further analysis.

Statistical analysis was performed with GraphPad Prism ver. 5.0b for Mac OS X (GraphPad Software, San Diego, CA, USA) and SPSS ver. 20.0 (SPSS Inc., Chicago, IL, USA).

#### Validity of the POMS

From a secure research database (Microsoft Access, Microsoft Corporation, Redmond, USA), patients who had POMS assessments on day 3 and 5 postoperatively, and who had undergone elective major abdominal surgery meeting the inclusion criteria above, were identified. POMS data was then analyzed against hospital LOS. This represents the criterion validity of the POMS, with LOS being used as a surrogate for morbidity.

## Results

### Inter-rater agreement of the POMS

Fifty-one patients were recruited to the study giving a total of 263 individual POMS assessments. Overall the presence or absence of morbidity as defined by the POMS showed a 97.7% agreement between observers, with a κ coefficient of 0.912 (95% CI: 0.842 to 0.982), giving near perfect agreement (Table 2).

**Table 2:** Inter-rater correlation and percentage agreement of the presence or absence of morbidity as measured by the POMS

	**Day 1 (n = 51)**	**Day 2 (n = 51)**	**Day 3 (n = 51)**	**Day 4 (n = 46)**	**Day 5 (n = 40)**	**Total (n = 263)**
Percentage agreement (%)	100	100	100	95.7	95	97.7
κ correlation coefficient (95% CI)	N/A	1.0 (1.0-1.0)	1.0 (1.0-1.0)	0.873 (0.760-1.0)	0.895 (0.760-1.0)	0.912 (0.842-0.982)

When observers scored all patients the same the hypothetical probability of chance could not be calculated, and are labeled N/A.

The percentage agreements and κ coefficients for individual POMS domain on days 1, 3, 5 and cumulatively, are shown in Table 3. Overall percentage agreement was high for the wound domain at 99.2%. However, there was no evidence of agreement beyond that expected by chance alone, and κ was estimated at near zero. Fair agreement was seen in the hematological domain, substantial agreement in the gastrointestinal and neurological domains, and almost perfect agreement in the remaining 6 domains [6].

**Table 3:** Inter-rater correlation and percentage agreement for individual domains of the POMS on days 1, 3, 5 and cumulatively

	**Day 1 (n = 51)**		**Day 3 (n = 51)**		**Day 5 (n = 40)**		**Total (n = 263)**	
	**Percentage agreement**	**κ**	**Percentage agreement**	**κ**	**Percentage agreement**	**κ**	**Percentage agreement**	**κ**
Cardiovascular	94.1	0.876 (0.740-1.0)	100	1.0 (1.0-1.0)	97.5	0.655 (−0.012-1.0)	96.6	0.828 (0.718-0.939)
Gastrointestinal	88.2	0.555 (0.221-0.890)	84.3	0.651 (0.443-0.858)	87.5	0.658 (0.377-0.938)	87.5	0.749 (0.669-0.829)
Hematological	80.4	0.069 (−0.448-0.587)	100	1.0 (1.0-1.0)	100	N/A	95.4	0.378 (0.035-0.722)
Infectious	98.0	0.929 (0.793-1.0)	98.0	0.935 (0.810-1.0)	95.0	0.804 (0.539-1.0)	98.1	0.938 (0.884-0.992)
Neurological	96.1	0.480 (−0.227-1.0)	96.1	0.779 (0.479-1.0)	97.5	0.655 (−0.012-1.0)	97.0	0.717 (0.524-0.910)
Pain	100	1.0 (1.0-1.0)	86.3	0.71 (0.511-0.90)	90.0	0.610 (0.247-0.973)	93.5	0.869 (0.809-0.929)
Pulmonary	100	1.0 (1.0-1.0)	94.1	0.870 (0.727-1.0)	95.0	0.890 (0.742-1.0)	96.2	0.906 (0.852-0.960)
Renal	98.0	0.658 (−0.007-1.0)	96.1	0.897 (0.757-1.0)	92.5	0.845 (0.677-1.0)	95.1	0.869 (0.802-0.936)
Wound	98.0	0.0 (−1.0-1.0)	100	N/A	100	N/A	99.2	−0.004 (−1.0-1.0)

Some κ coefficients could not be calculated as all observations occurred in a single domain, and are labeled N/A.

### Validity of the POMS

**Figure 1:**
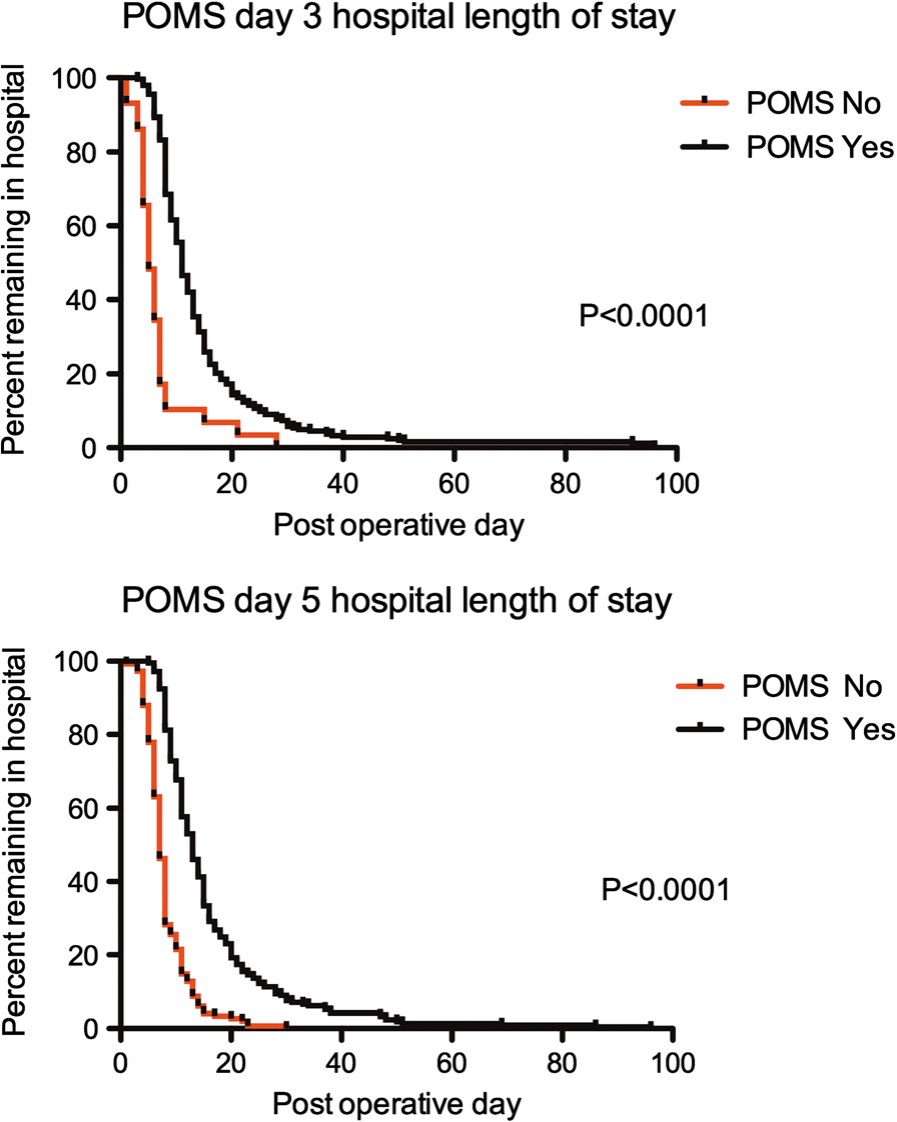
Kaplan-Meier curves for hospital LOS based upon the presence or absence of POMS defined morbidity on postoperative days 3 and 5.

POMS assessments were analyzed for postoperative day 3 (n = 258) and postoperative day 5 (n = 362) (Table 4). This data was obtained from the prospective inter-rater reliability study previously described, and three other studies performed in the same institution in which POMS data was prospectively obtained [7,8]. The median LOS for the cohort was 11 days (IQR: 8 to 15 days). The absence or presence of morbidity measured by the POMS was associated with a hospital LOS of 6 (IQR: 4 to 7) *vs.* 11 (IQR: 8 to 15) days when performed on postoperative day 3 (*P* <0.0001), and 7 (IQR: 6 to 10) *vs.* 13 (IQR: 9 to 19) days when performed on postoperative day 5 (*P* <0.0001). Kaplan-Meier analysis for the POMS on postoperative days 3 and 5 and hospital LOS are shown in Figure 1.

**Table 4:** Number and percentage of patients in each surgical specialty analyzed in the assessment of the validity of the POMS

	**Day 3**	**Day 5**
	**n (%)**	**n (%)**
Colorectal	213 (82.6%)	260 (71.8%)
Vascular	8 (3.1%)	31 (8.6%)
Urology	35 (13.5%)	61 (16.8%)
Upper gastrointestinal	2 (0.8%)	10 (2.8%)
Total	258	362

The incidence of morbidity in different domains for postoperative days 3 and 5 are shown in Table 5, with renal, pulmonary, gastrointestinal and pain morbidity having the highest incidence on both days.

**Table 5:** Incidence of morbidity by POMS domain on postoperative days 3 and 5

	**Incidence of morbidity (%)**	
**Domain**	**Day 3**	**Day 5**
Neurological	10.0	3.2
Hematological	3.8	1.2
Pain	70.5	28.7
Gastrointestinal	53.6	31.9
Wound	0	1.2
Cardiovascular	9.2	6.1
Pulmonary	54.7	42.1
Infectious	157	17.4
Renal	46.7	26.3

**Figure 2:**
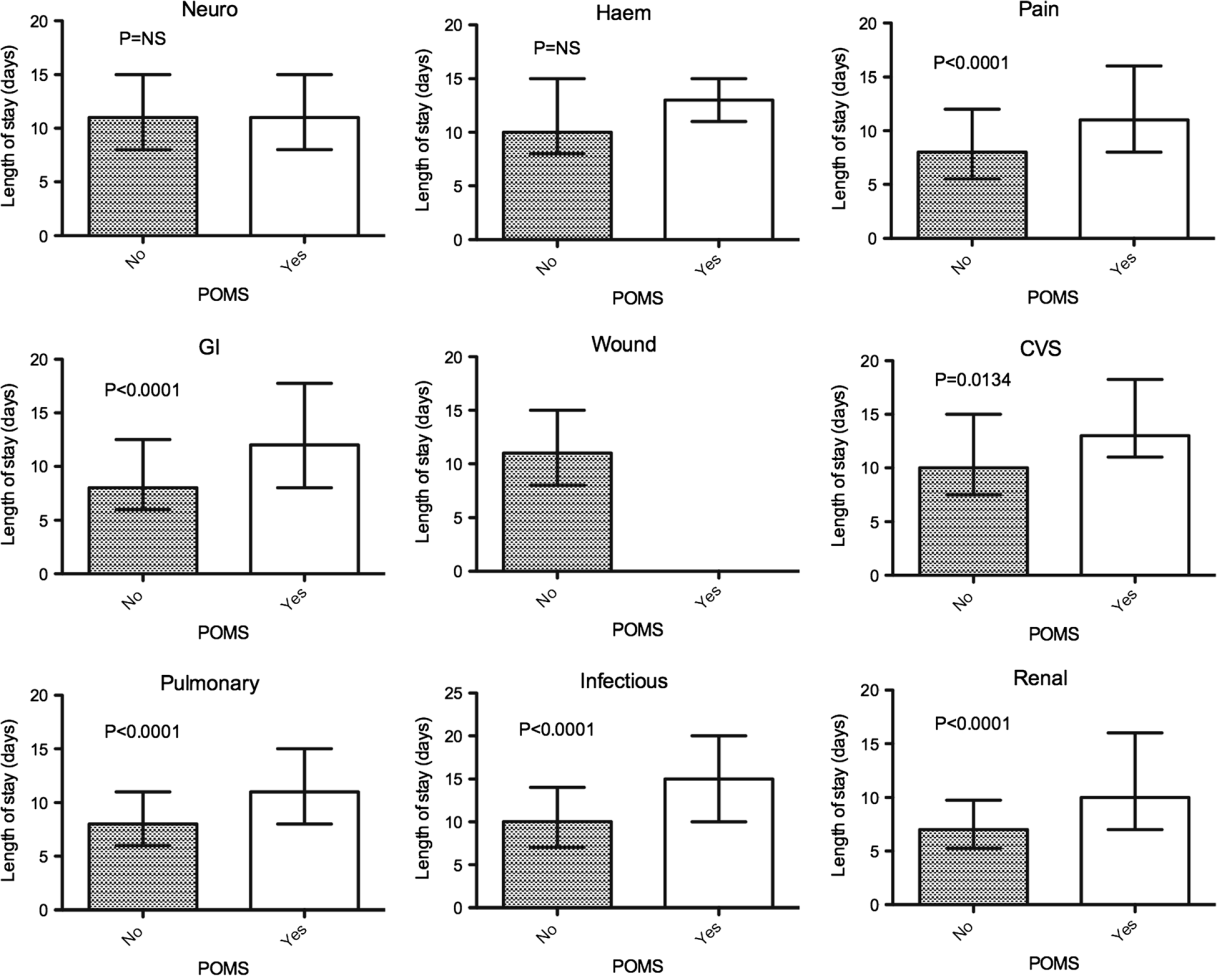
**Hospital LOS for the presence or absence of morbidity by individual POMS domain type on postoperative day 3.** Data is reported as median with interquartile range.

**Figure 3:**
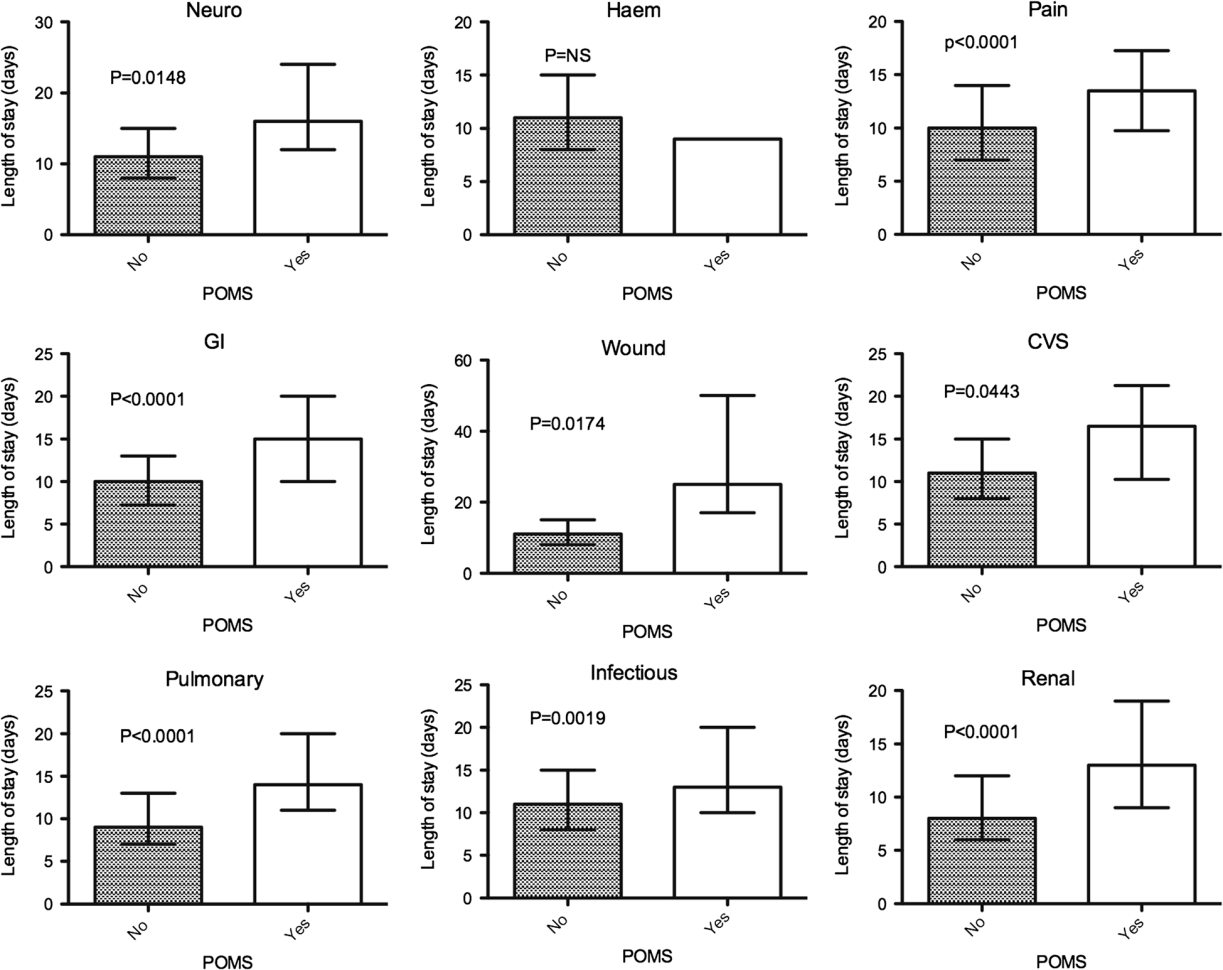
**Hospital LOS for the presence or absence of morbidity by individual POMS domain type on postoperative day 5.** Data is reported as median with interquartile range.

The relationship between morbidity in individual domains on postoperative days 3 and 5, and median hospital LOS is shown in Figures 2 and 3. On day 3, the presence of morbidity in seven out of the nine domains was associated with a longer hospital LOS, and on day 5 morbidity in all domains, with the exception of hematological morbidity, was also associated with a longer LOS. On multivariate regression analysis only the presence of morbidity in the pulmonary domain on both days 3 and 5 was statistically significant in explaining the variations in hospital LOS (*P* <0.0001).

The presence of any morbidity on postoperative day 5 conferred an OR of 11.9 (95% CI: 5.02 to 28.31) for a prolonged hospital LOS, defined as greater than the 3^rd^ interquartile for the cohort, but no predictive ability was conferred to the same analysis on day 3 (OR: 3.64; 95% CI: 0.83 to 15.92). ROC analysis by domain and day for LOS is shown in Table 6, with significant differences seen in the AUC on days 3 and 5 for the cardiovascular and gastrointestinal domains.

**Table 6:** ROC analysis by POMS domain and postoperative day measured for LOS

**Domain**	**Day**	**AUC (95% CI)**	**Z**
Cardiovascular	3	0.69 (0.60-0.76)	2.699*
	5	0.46 (0.29-0.60)	
Gastrointestinal	3	0.70 (0.63-0.76)	2.355*
	5	0.58 (0.50-0.65)	
Hematological	3	0.70 (0.60-0.80)	0.307
	5	0.65 (0.33-0.97)	
Infectious	3	0.70 (0.62-0.78)	1.808
	5	0.59 (0.50-0.68)	
Neurological	3	0.60 (0.46-0.73)	1.311
	5	0.73 (0.58-0.87)	
Pain	3	0.73 (0.65-0.80)	1.863
	5	0.63 (0.56-0.70)	
Pulmonary	3	0.75 (0.69-0.81)	0.814
	5	0.71 (0.65-0.76)	
Renal	3	0.50 (0.42-0.57)	0.201
	5	0.48 (0.40-0.57)	
Wound	3	N/A	N/A
	5	0.52 (0.37-0.67)	

*Statistically significant difference between the AUC.

POMS defined morbidity was absent in 9.7% of patients on day 3 and 41.2% of patients on day 5 despite them still receiving inpatient care.

## Discussion

This study shows that the POMS is both a reliable and valid measure of short-term postoperative morbidity in patients undergoing major abdominal surgery. The main strength of this paper is that it studies a homogenous group of subjects all of whom underwent major abdominal surgery, and hence could reliably be expected to have a similar pattern of postoperative morbidity. The study also examined the reliability of the survey when administered by novice researchers unfamiliar with the POMS, allowing an assessment of its potential use as an outcome measure in both research and clinical practice, where it would be required to be administered by a spectrum of health professionals of varying experience.

Overall the inter-rater reliability of POMS-defined morbidity showed high percentage agreement, and near perfect correlation. Reliability within POMS domains showed varying results, with the lowest percentage agreement seen in the gastrointestinal domain, however the κ correlation coefficient still showed a substantial agreement. A similar result was also shown in the only other validation of the POMS [9], in which the gastrointestinal domain showed the weakest correlation between observers, suggesting that the criteria may need refining to give it greater clinical utility. In the study by Grocott *et al*. [9] the majority of the items within the POMS domains showed a perfect correlation, however this was not the case in the present study. The reason for the decreased correlation may be due to experienced research nurses having administered the survey in the previous work, whilst we chose specifically to assess the reliability of the POMS when it was administered by novices to its use. Despite this, POMS, as it currently stands, could be used as an outcome measure without specific training in its use.

There are a number of apparent discrepancies in the reliability of individual domains as only fair correlation was seen in the hematological domain, and no correlation was seen in the wound domain despite good percentage agreement between observers. The use of the κ coefficient in this setting unfairly describes the reliability of the POMS as κ is affected by the prevalence of the findings it is describing. When the reported prevalence is low, as was the case in the hematological and wound domains, then κ may not be a reliable measure of correlation as illustrated by the high agreement but poor correlation [10].

As the POMS cannot be used as a scale due to its low internal consistency [9], the presence of any morbidity recorded by the POMS must be associated with an increased hospital LOS in order for it to be valid. The presence of morbidity registered by the POMS at both postoperative days 3 and 5 was associated with a longer hospital LOS, and morbidity at day 5 was associated with an eightfold increase in patients having a prolonged hospital LOS (greater than the 3^rd^ interquartile for the cohort). These observations support the predictive ability of the POMS, but also suggest additional utility for the survey in the context of resource utilization due to its ability to predict those who will remain in hospital for longer.

Grocott *et al.* found that on postoperative day 5 the presence of morbidity within 5 of the 9 domains was associated with a longer hospital LOS; however, we observed this in an additional 3 domains. This may reflect the significant differences in the two populations studied, with the previous cohort consisting of almost two thirds orthopedic patients undergoing peripheral surgery, whilst this study contained a more homogenous cohort all of whom underwent major abdominal surgery. In addition, it also appears that a number of domains are associated with a longer hospital LOS when the POMS is administered earlier at postoperative day 3, allowing a more prompt assessment of significant morbidity that affects hospital LOS and strengthening its validity as an outcome measure.

The pattern of morbidity seen in this study is similar to that previously described [5,9], although higher incidences of pain and pulmonary related morbidity, but lower incidences of gastrointestinal morbidity related morbidity were seen. This is surprising given the nature of the cohort, but a possible explanation for this reduction in gastrointestinal morbidity in a series that has undergone major abdominal surgery may due to differences in postoperative analgesia (epidural *versus* intravenous opiates), or the adoption of enhanced recovery protocols aimed at minimizing morbidity.

There are a number of limitations to this study, primarily the small numbers in the reliability assessment of the POMS; however, the overall number of observations was acceptable. The cohort is not truly homogenous, and although all subjects underwent major abdominal surgery, the inclusion of vascular and urology patients, who may have a different prevalence and pattern of morbidity to those undergoing colorectal surgery, may limit the application of results to this population. In addition we did not assess patients with no POMS defined criteria for potentially important morbidity not captured by the survey. It is possible that this group remained hospitalized due to significant postoperative morbidity rather than social issues or delays in the discharge process. However, it has previously been observed that patients remaining in hospital with no POMS defined morbidity showed no evidence of any other morbidity measured by different means [5,9].

There are also a number of limitations of the POMS itself as many of the domains describe what is considered routine therapy following major surgery (postoperative oxygen, antibiotics, catheterization), however, by postoperative days 3 and 5, these routine therapies should have been ceased, particularly in the context of enhanced recovery programs. The lack of internal consistency of POMS, meaning that it cannot be treated as a scale, only allows the POMS to identify the presence of morbidity but not to measure its severity, a tool that would be inherently useful when measuring postoperative outcomes. Further modification of the POMS may allow the development of a survey that measures a single construct, and hence can be used to measure the severity of morbidity. The validity of the POMS in different and specific surgical cohorts, for example vascular and urology, also needs to be explored as the patterns of morbidity are often different. However, it appears to be both valid and reliable in this group.

Accurate and meaningful reporting of outcomes in clinical trials is essential; however, it has been acknowledged that reporting of surgical adverse events in the health system is inconsistent [11]. The underlying problem remains the lack of standardization of reported outcome measures in terms of timescale, the specific measurement, and the definition of that same measurement. The standardization of a core set of outcome measures would allow for more effective comparison of interventions, allow appropriate outcome measures to be collected and reported in the correct way, aid meta-analysis and sample size calculations, and simplify the design of trials for researchers. An additional benefit of reporting a set of core outcome measures would be the reduction of outcome reporting bias. Outcome reporting bias can be defined as the selection for publication of a subset of the originally reported outcome variables based on the results [12].

The POMS is essentially a composite outcome measure, consisting of various domains in which those with multiple items can be collapsed so that each domain becomes a binary outcome. The primary outcome measure can then be considered a composite of the binary outcomes for each domain. A composite outcome measure such as the POMS has a number of inherent advantages over a single clinical endpoint, particularly in clinical trials. Major complications as a core outcome measure have a relatively low event rate; however, the composite measure which combines outcomes increasing the overall incidence of events, improves the power of a study for a set number of participants, or reduces the number of participants required to achieve a preset power. The multi-component nature of composite measures allows researchers the opportunity to be able to describe the disease, or its process, more effectively, particularly in complex states that affect multiple systems such as the inflammatory response and the associated morbidity following major abdominal surgery. The components of a composite measure should describe the outcome of interest, and the POMS was designed by clinicians to reflect clinically important issues that caused patients to remain in hospital after major surgery. Ideally the components should have similar frequency, treatment effects and severity [13,14], however, this is practically difficult in complex disease processes, and can be compensated for by modern statistical methodology, which also allows not only an overall treatment effect to be detected from the composite, but also the effect of the treatment on the individual components [15].

Various statistical methodologies are available to clinically utilize the POMS as a core outcome measure following major abdominal surgery. The composite can be collapsed to a single outcome; however, high frequency items are then over-weighted at the expense of less frequently occurring items that may be more clinically important. Alternatively, a count of events may be employed; however, once again, this can be difficult to interpret unless all events are of an equal severity. Multivariate generalized estimating equation (GEE) methods [16,17] are less affected by issues of unequal severity and frequency [15] and can be described in terms of a common effect GEE in which a treatment effect is estimated across all components of the composite, or an average relative effect GEE in which a treatment effect is estimated for each component. In terms of the POMS as the common effect GEE methods are still biased by events that occur with a higher frequency, a situation that arises in this survey with the gastrointestinal and pain domains having a high frequency, whereas morbidity in the wound domain rarely occurs, the average relative effects GEE is the more appropriate statistical tool to utilize as it compensates for this effect. In addition clinically derived weights for the importance of the various domains can be applied to this model increasing its clinical utility, although these must be assigned prior to data analysis.

Further work is required to simplify the POMS, and to determine which domains and items have poor predictive ability, allowing modification of the survey with additional items with the view to constructing a survey that has acceptable internal consistency, and hence can be used as a scale. Additional consideration is also required as to the potential of weighting individual domains based on perceived clinical importance.

## Conclusions

The POMS is reliable, easy to use and shows predictive validity when compared to hospital LOS, and also predicts those subjects who will have a prolonged hospitalization. It has the potential not only to be used as a core outcome measure following major surgery, but also as a tool to aid resource utilization.

## Abbreviations

GEE: Generalized estimating equation; LOS: Length of stay; POMS: Postoperative Morbidity Survey.

## Competing interests

The authors declare that they have no competing interests.

## Authors’ contributions

SJD contributed to the study design, conduct of study, data analysis and manuscript preparation. JF and JD participated in data analysis, conduct of the study and manuscript preparation. R JTW helped with the study design, data analysis and manuscript preparation. SJH contributed to study design and manuscript preparation. VA helped with data analysis and manuscript preparation. All authors read and approve the final manuscript.
